# The expanding horizon of neurotechnology: Is multimodal neuromodulation the future?

**DOI:** 10.1371/journal.pbio.3002885

**Published:** 2024-10-28

**Authors:** Silvestro Micera, Guglielmo Foffani

**Affiliations:** 1 Bioelectronics Area and MINE Laboratory, The BioRobotics Institute, and Department of Excellence in Robotics and AI, Scuola Superiore Sant’Anna, Pisa, Italy; 2 Bertarelli Foundation Chair in Translational Neuroengineering, Neuro-X Institute, School of Engineering, Ecole Polytechnique Federale de Lausanne (EPFL), Lausanne, Switzerland; 3 Modular Implantable Neuroprostheses (MINE) Laboratory, Università Vita-Salute San Raffaele, Milan, Italy; 4 HM CINAC (Centro Integral de Neurociencias Abarca Campal), Hospital Universitario HM Puerta del Sur, HM Hospitales, Madrid, Spain; 5 Hospital Nacional de Parapléjicos, SESCAM, Toledo, Spain; 6 CIBERNED, Instituto de Salud Carlos III, Madrid, Spain; 7 Instituto de Investigación Sanitaria HM Hospitales, Madrid, Spain

## Abstract

The clinical applications of neurotechnology are rapidly expanding, and the combination of different approaches could be more effective and precise to treat brain disorders. This Perspective discusses the potential and the challenges of ‘multi-modal neuromodulation’, which combines modalities such as electrical, magnetic and ultrasound stimulation.

## Teaser

The clinical applications of neurotechnology are rapidly expanding, and the combination of different approaches could be more effective and precise to treat brain disorders. This Perspective discusses the potential and challenges of “multimodal neuromodulation,” which combines modalities such as electrical, magnetic, and ultrasound stimulation.

Neurotechnology is rapidly evolving from its origins in basic brain mapping and diagnostic tools into an innovative field with real, transformative clinical applications. Recent advancements have made neurotechnology more clinically viable, allowing for effective treatment and rehabilitation options for various neurological and psychiatric disorders. These technologies involve different techniques, such as electrical stimulation, magnetic fields, and emerging modalities like ultrasound, optoelectronics, and optogenetics. Such approaches have been successfully implemented in procedures such as deep brain stimulation (DBS) [1], epidural electrical stimulation (EES) [2], and transcranial magnetic stimulation (TMS) [3], which have moved beyond the lab to become standard clinical tools for treating disorders like Parkinson’s disease, epilepsy, spinal cord injury, and depression. Similarly, cochlear implants and other neural prosthetics have revolutionized the treatment of sensory deficits. Artificial limbs are more and more able to be fully integrated into the nervous system [4]. However, as our understanding of the nervous system grows, it has become clear that single-modality approaches like electrical or magnetic stimulation may have limitations. The nervous system is a highly complex, multidimensional system that involves various physical processes—electrical signaling, chemical interactions, mechanical movements of cells, and even light-sensitive proteins. Thus, there is growing interest in leveraging multiple physical modalities—not just electrical or magnetic but also mechanical, optical, and even genetic modulation techniques—to better target and treat brain disorders. We refer to this next frontier of neurotechnology as “multimodal neuromodulation”.

Each neuromodulation technique has strengths and weaknesses, and these limitations highlight the need for multimodal approaches. For example, invasive electrical stimulation (e.g., DBS, vagus nerve stimulation, and epidural electrical stimulation) is precise and allows deep neural access, but requires surgery, with its associated risks and unintended side effects. Thus, noninvasive techniques are often used, including noninvasive electrical stimulations [5] such as transcranial direct current stimulation, transcranial alternating current stimulation, transcranial random noise stimulation and, more recently, temporal interference stimulation [6]. While these techniques can noninvasively reach cortical (and deeper) targets, their precision and reliability are limited by the distortion of the electrical field by biological tissues, which makes it challenging for the current to reach the intended target. Conversely, TMS [3] is also noninvasive and can be relatively more focal, but its depth of penetration is limited. Its effects are also sometimes inconsistent across patients. Recent developments suggest that TMS in the kHz range, called kHz transcranial magnetic perturbation (kTMP), can also modulate cortical excitability [7]. Meanwhile, transcranial static magnetic field stimulation (tSMS) [8] is not distorted by biological tissues and is fully portable, which is convenient for long-term home-based treatments [9], but its depth and focality are limited.

Given the limitations of electrical and magnetic stimulation, alternative methods for neuromodulation have also been developed. For example, ultrasound neuromodulation has shown potential for precision targeting of cortical and deep brain structures [10]. Ultrasounds can interact with mechanosensitive channels in cells, potentially influencing blood–brain barrier permeability and neural excitability, but the effects of ultrasounds on neural tissue are still not fully understood, and their safety and efficacy require further investigation. Alternatively, neurons can be stimulated using photons [11]. Optogenetics uses genetic modifications to make neurons responsive to light, offering cell-type precision that is widely employed for selective manipulations in basic neuroscience, but has limited clinical viability due to the ethical and technical challenges of gene therapy [12]. Optoelectronic neuromodulation promises to deliver non-genetic stimulation with similar cellular precision, but requires invasive procedures to implant light-sensitive components [13]. Other developments are ongoing.

**Fig 1:**
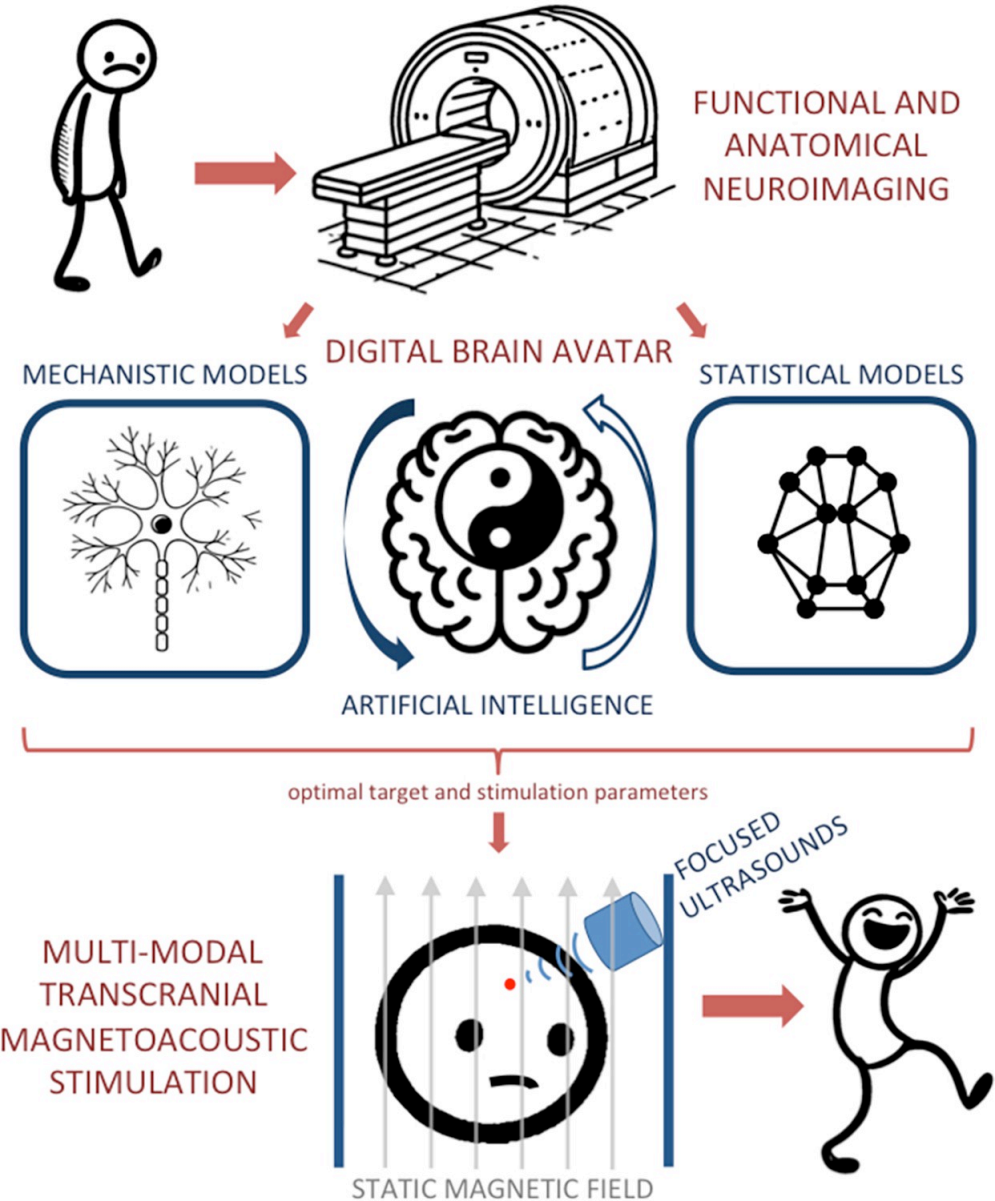
Potential implementation of multimodal neuromodulation. Schematic depicting a possible future scenario for a patient that is suffering from a neurological or psychiatric disorder and is a candidate for brain stimulation therapy: (i) based on neuroimaging of the specific patient and of previous patients, artificial intelligence builds a digital avatar as a multi-scale replica of the patient’s brain, integrating bottom-up mechanistic modeling and top-down statistical modeling; (ii) the artificial intelligence then optimizes and personalizes a highly precise but noninvasive multimodal neuromodulation therapy on the patient’s avatar (best target, optimal parameters, etc.); (iii) only the optimal personalized stimulation is prescribed to the patient and provides maximal therapeutic benefit. In this example, the specific implementation of multimodal neuromodulation is transcranial magneto-acoustic stimulation (TMAS), which has already been conceptualized and tested in computational models and animal experiments, and awaits to be translated to humans [14].

Each modality taps into different principles of physics—electrical currents, magnetic fields, acoustic waves, and photons—and each engages with the brain in distinct ways. The complexity of neural circuits and the heterogeneity of neural diseases suggest that combining these approaches could offer significant therapeutic benefits. Multimodal neuromodulation refers to the simultaneous or sequential use of multiple physical modalities to influence brain activity. This could mean combining electrical, magnetic, and ultrasound stimulation in a single treatment protocol, or even integrating optical and genetic approaches for more precise control. Multimodal neuromodulation could in principle be personalized with artificial intelligence, using neuroimaging data to build a digital twin of the patient’s brain, where the stimulation is optimized in silico to maximize clinical benefit. A conceptual example is shown in Fig 1.

Such a paradigm shift may offer a few key advantages. Firstly, increased precision and specificity: by integrating different modalities, multimodal neuromodulation could allow for more targeted stimulation of specific brain circuits. For example, electrical stimulation or tSMS could be used to engage broad areas of the brain, while ultrasound or light-based techniques precisely modulate small groups of neurons. Secondly, it could allow comprehensive control over neural activity: electrical, magnetic, and optical signals influence the brain in different ways. Combining these modalities could offer more comprehensive control over how neurons and other cell types behave, which could lead to more effective therapies. In addition, there would be greater potential for noninvasive treatments: multimodal approaches may allow for noninvasive treatment options that rival the efficacy of invasive ones like DBS. For example, focused ultrasound is already being used for non-surgical brain lesions, and optogenetics could potentially be delivered noninvasively through the bloodstream in the future. By using combinations of noninvasive modalities, it may be possible to achieve outcomes similar to those of more invasive procedures, improving patient safety and comfort. Finally, there may be benefits from synergistic effects: some forms of neuromodulation may work better in combination than alone. As a speculative example, ultrasound may make neurons more responsive to electrical or magnetic stimulation. Combining optical stimulation with ultrasound could target specific neuronal subpopulations while influencing their local microenvironment. The synergistic effects of these modalities could open up new avenues for treating diseases that are currently difficult to address with single-modality therapies.

Despite its promise, multimodal neuromodulation faces several technological and ethical challenges. Integrating multiple modalities requires significant advancements in engineering, as well as a deep understanding of how different physical modalities interact with the brain. This will involve the development of new devices that can deliver combinations of stimuli in a safe, precise, and controlled manner. Moreover, while some approaches like electrical and magnetic stimulation are relatively well understood, others, like ultrasound and optogenetics, are still in relatively early stages of development and require further research to fully understand their long-term effects.

The future of neurotechnology is likely to be shaped by the convergence of various physical modalities into multimodal neuromodulation systems. As scientists and engineers continue to develop and refine these technologies, it will become increasingly possible to treat a wider range of neurological and psychiatric disorders with greater precision. This could revolutionize the treatment of conditions like epilepsy, depression, chronic pain, and neurodegenerative diseases, offering hope to millions of patients worldwide. However, the path forward will require a concerted effort to overcome the technical, ethical, and regulatory hurdles that lie ahead. As multimodal neuromodulation becomes more clinically viable, it will be essential to ensure that these technologies are used safely, effectively, and equitably. Only then will the full potential of this revolutionary approach be realized, ushering in a new era of precision neurotechnology.
